# Publish, not perish: Introducing *Experimental Hematology & Oncology*

**DOI:** 10.1186/2162-3619-1-1

**Published:** 2012-03-26

**Authors:** Zihai Li, Chung-Tsen Hsueh, Delong Liu

**Affiliations:** 1 Department of Microbiology & Immunology, Medical University of South Carolina, Charlston, SC 29425, USA; 2 Loma Linda University Medical Center, Loma Linda, CA 92354, USA; 3 New York Medical College, Valhalla, NY 10595, USA

## Abstract

As a scientific discipline, medicine can only be advanced by experimentation. Experimentation could either validate or refute a hypothesis. Unfortunately, today's publication climate strongly favors publication of positive research findings, especially with clinical trials. *Experimental Hematology & Oncology *(*e*HO) is a new open access online journal that emphasizes preclinical, patient-oriented and translational aspects of research. The journal differentiates from others in the field by making a deliberate effort in publishing clinical trials with "negative" results and basic science studies with provocative findings. The focus of the peer-review mechanism for *e*HO will be on the technical merit of the study and not on demanding a long list of additional experiments that hinders rapid information dissemination.

## Introduction

Medicine is moving rapidly from experience-based practice to more scientific and evidence-based endeavor. Sharing information in real time not only makes scientific sense but also could be life-saving. There have been instances of a number of drugs whose adverse health effects were not made publicly available for several years after the drug had been released onto the market. It is our opinion that many lives could be saved by publishing information such as this more widely and in a more timely manner. For example, Gemtuzumab ozogamicin (Mylotarg) is a calicheamicin-linked monoclonal antibody against CD33, approved to treat relapsed acute myelogenous leukemia in 2000. It was voluntarily withdrawn from the market in June 2010 due to the possibility that the drug may increase patient death and provided no additional benefit over conventional therapies [1]. However, the related study (SWOG S0106) results have not been officially published to date.

## All science that is fit to print

In the spirit of open access and rapid dissemination of information, we are proud to launch *e*HO. *e*HO is an open access, peer-reviewed online journal that encompasses all aspects of hematology and oncology with emphasis on preclinical, patient-oriented and translational aspects of research. *e*HO aims to publish original work, hypothesis, commentaries and timely reviews in the field of hematology and oncology.

The intention of this journal is not to compete, but complement the existing journals in the field. With open access, rapid turnaround time from submission to publication and online live comments from readers, *e*HO will strive to be the hub for disseminating new knowledge and discussing controversial topics for both basic scientists and busy clinicians in the fields of hematology and oncology.

Authors will be pleased to know that the philosophy of this journal is to leave the judgment of the scientific interest of the work to the readers. Instead, the editorial and publication team [2] will focus primarily on publishing original work that provides a useful contribution to the scientific knowledge in the field, while ensuring the highest ethical standards. Once published, articles are freely and universally available online, meaning a higher potential readership for the research.

For the vast majority of physicians and scientists, this will mean that you do the science and discovery, *e*HO does the publishing! The winners will be the patients and the scientific community!
